# Dosimetric analysis of MR‐LINAC treatment plans for salvage spine SBRT re‐irradiation

**DOI:** 10.1002/acm2.13752

**Published:** 2022-08-25

**Authors:** Eun Young Han, Debra N. Yeboa, Tina M. Briere, Jinzhong Yang, He Wang

**Affiliations:** ^1^ Department of Radiation Physics The University of Texas MD Anderson Cancer Center Houston Texas USA; ^2^ Department of Radiation Oncology The University of Texas MD Anderson Cancer Center Houston Texas USA

**Keywords:** dosimetry, MR‐Linac, spine SBRT

## Abstract

**Purpose:**

We investigated the feasibility of thoracic spine stereotactic body radiotherapy (SBRT) using the Elekta Unity magnetic resonance‐guided linear accelerator (MRL) in patients who received prior radiotherapy. We hypothesized that Monaco treatment plans can improve the gross tumor volume minimum dose (GTVmin) with spinal cord preservation and maintain consistent plan quality during daily adaptation.

**Methods:**

Pinnacle clinical plans for 10 patients who underwent thoracic spine SBRT (after prior radiotherapy) were regenerated in the Monaco treatment planning system for the Elekta Unity MRL using 9 and 13 intensity‐modulated radiotherapy (IMRT) beams. Monaco adapt‐to‐position (ATP) and adapt‐to‐shape (ATS) workflow plans were generated using magnetic resonance imaging with a simulated daily positional setup deviation, and these adaptive plans were compared with Monaco reference plans. Plan quality measures included target coverage, Paddick conformity index, gradient index, homogeneity index, spinal cord D_0.01cc_, esophagus D_0.01cc_, lung V10, and skin D_0.01cc_.

**Results:**

GTVmin values from the Monaco 9‐beam and 13‐beam plans were significantly higher than those from Pinnacle plans (*p* < 0.01) with similar spinal cord dose. Spinal cord D_0.01cc_, esophagus D_0.01cc_, and lung V10 did not statistically differ among the three plans. The electron‐return effect did not induce remarkable dose effects around the lungs or skin. While in the ATP workflow, a large increase in GTVmin was observed at the cost of a 10%–50% increase in spinal cord D_0.01cc_, in the ATS workflow, the spinal cord dose increase was maintained within 3% of the reference plan.

**Conclusion:**

These findings show that MRL plans for thoracic spine SBRT are safe and feasible, allowing tumor dose escalation with spinal cord preservation and consistent daily plan adaptation using the ATS workflow. Careful plan review of hot spots and lung dose is necessary for safe MRL‐based treatment.

## 1 INTRODUCTION

Fractionated spine stereotactic body radiotherapy (SBRT) has been shown to be an important radiotherapy option for spinal metastases in patients who have had prior radiotherapy or for whom large setup uncertainty is expected.^1^, ^2^, ^3^ Improved therapeutic benefit can be attained with a minimum dose to the GTV (GTVmin) of 21 Gy in three fractions in the treatment naïve setting.^4^ In the salvage re‐irradiation setting, the highest possible GTVmin close to this value, while prioritizing first organs at risk (OARs), is the goal. Because re‐irradiation is dependent on the unique nature of the prior radiotherapy dose and fractionation, the different OARs in field, and time since radiation, the SBRT plans are driven primarily by the combined dose to the OARs or allowable re‐irradiation dose to the OARs while attempting to maintain adequate GTVmin dose.

Also, spine SBRT requires a complex treatment plan with highly conformal dose distribution for the target and a steep dose fall‐off near the spinal cord to meet the spinal cord dose constraint, which is often as low as a 10‐Gy dose maximum in patients who have undergone previous radiotherapy.^4^ If the distance between the GTV (27 Gy) and the spinal cord (10 Gy) is less than 5 mm, the dose fall‐off from the GTV must be >3.0 Gy/mm, and therefore, GTV coverage is intrinsically limited by the spinal cord dose constraints, possibly resulting in less optimal tumor control.^5^

In conventional linear accelerator (linac)‐based treatment, the spinal cord is not visible in cone beam computed tomography‐based image‐guided radiotherapy, and therefore the spinal canal is often used as a surrogate anatomy for the pretreatment patient setup for spine SBRT. The bulky spinal canal used in dose constraints may protect the true spinal cord, but it also limits target coverage when the target abuts the spinal cord. In addition, a study by Oztek et al. showed that the spinal cord can move with respect to rigid bones, resulting in detrimental dose effects to the spinal cord.^6^ Differences in pretreatment patient setup have been observed when aligning to the spinal canal as a surrogate instead of the spinal cord.^7^

Conversely, the use of a MR–guided linac (MRL) is expected to enhance spine SBRT because of improved soft tissue contrast with the onboard magnetic resonance imaging (MRI), online daily spinal cord delineation, and daily plan adaptation. Target dose escalation near the spinal cord through better soft tissue visualization would fill a need for improved tumor control while safely sparing the spinal cord adjacent to the tumor. In the Elekta MRL, the irradiation geometry (7.2‐mm width multileaf collimator [MLC]), beam configuration (143.5‐cm source‐axis distance [SAD]), and 1.5‐T magnetic field greatly deviate from the conventional linacs.^8^ Feasibility studies have reported clinically acceptable MRL plan quality for SRS/SBRT of brain metastases, lung cancer, pelvic lymph nodes, and gastrointestinal cancer.^9^, ^10^, ^11^, ^12^

Lee et al. reported a statistically significant increase in target coverage in the Monaco treatment planning system (TPS) compared with other TPSs for conventional Linac treatment plans.^13^ In our previous study, which showed a potential for spine SBRT on MRL through planning and delivery using an anthropomorphic spine phantom,^8^ we found that the Monaco TPS can generate a plan for MRL similar to those generated by conventional TPSs. However, the mean spinal cord dose was increased in the adapted plans by up to 8.0% compared with the reference plans during the adapt‐to‐position (ATP) workflow. It has also been reported that the electron return effect (ERE) can result in dose increases at the air‐tissue interface, such as in the lungs and skin.^9^, ^14^, ^15^ In addition, inferior target conformality and higher low‐dose volumes compared with conventional linac‐based plans were reported by other authors.^14^, ^16^, ^17^ Therefore, it is necessary to thoroughly investigate dosimetric aspects of MRL treatment plans for thoracic spine SBRT, as well as the stability of online adaptive plans for MRL.

The purpose of the current dosimetric study was to investigate the feasibility of thoracic spine SBRT on the Elekta Unity MRL in patients who have received previous radiotherapy. We hypothesized that use of the Monaco TPS can improve the GTVmin with similar target coverage and spinal cord dose while maintaining consistent plan quality during the daily adaptation process.

## 2 METHODS

### 2.1 Reference plan generation

Clinical treatment plans from 10 patients who underwent thoracic spine SBRT, originally generated on the planning CT in the Pinnacle TPS, were regenerated in the Monaco TPS for the Elekta Unity MRL. Optimization goals for targets included V100% ≥95% (at least 95% of the target to be covered by the prescription dose), maximum dose (D_0.01cc_) ≤120% of the prescription dose, and GTVmin ≥21 Gy. Dose constraints for previously irradiated spinal cords were V9Gy ≤1 cm^3^ (volume of the spinal cord receiving 9 Gy should be less than 1 cm^3^) and D_0.01cc_ ≤10 Gy or 14 Gy depending on prior treatment history and previously received dose to the spinal cord. Dose constraints for the previously irradiated esophagus were V14Gy ≤1 cm^3^ and D_0.01cc_ ≤21 Gy. Dose constraints were V10Gy ≤600 cm^3^ for lungs and V16Gy ≤10 cm^3^ and D_0.01cc_ ≤21 Gy for skin.

The calculation dose grid size was set to 2 mm for both CT‐based Pinnacle and Monaco plans. The plans in Monaco were optimized to yield the best possible plan but mainly focused on target coverage with the similar spinal cord dose as in the Pinnacle plans while meeting esophagus and skin dose constraints. The Monaco plans were then reviewed by the radiation oncologist.

**Pinnacle plans (v9.10)** were created for Varian TrueBeam STx using 6‐MV flattening‐filtered photon beams and 120 high‐definition MLC leaf pairs with 2.5‐mm thickness at the isocenter. Volumetric‐modulated arc therapy (VMAT) with four posterior quadrant arcs or step‐and‐shoot intensity‐modulated radiotherapy (IMRT) with nine posterior oblique gantry angles were employed. The collapsed cone convolution dose calculation method was used. A maximum of 100 segments per IMRT plan, a minimum of 2.0 monitor units (MUs) per segment, and a minimum segment area of 4.0 cm^2^ were allowed during plan optimization.

**The Monaco plans (v5.4)** were created for the Elekta Unity MRL (v1.0) with 7‐MV flattening filter free photon beams and 80 MLC leaf pairs with 7.2‐mm width at the isocenter. Two plans with nine posterior beams (same gantry angles as the Pinnacle plans ‐ 200, 220, 240, 260, 100, 120, 140, 160, and 180) and 13 encircling beams were generated to evaluate their impact on target conformity and stability during the plan adaptation process. Monte Carlo dose calculations were performed at <1% per calculation statistical uncertainty. A maximum of 100 segments per plan, a minimum of 2.0 MU per segment, and a minimum segment area of 2.0 cm^2^ were allowed during plan optimization.

### 2.2 Reference plan comparison

Monaco plans with nine beams and 13 beams were compared with the corresponding Pinnacle clinical plans. The plan quality index for comparison included target coverage (minimum, maximum, and mean doses), Paddick conformity index (PCI), gradient index (GI), homogeneity index (HI), D_0.01cc_ to the spinal cord, esophagus, and skin, V10Gy to the lungs and V50% to the body. The PCI was calculated as $\frac{{\mathrm{TV}}_{\mathrm{PIV}}^{2}}{\mathrm{TV}\;\times \;V100\%}$, where TV_PIV_ represents the target volume covered by the prescription isodose volume, V100% represents the patient volume covered by the prescribed dose, and TV is the target volume. The gradient index was calculated as $\frac{V50\%}{V100\%},$ where V50% represents the patient volume covered by 50% of the prescribed dose. The homogeneity index was calculated as $\frac{D2\%}{D98\%}$, where D2% represents the dose covered by 2% of the target volume.

### 2.3 Adaptive plan generation

In the ATP workflow, the daily MRI scan is used to identify a translational isocenter shift from the reference plan position. The plan is reoptimized (recalculated) on the CT (reference image) with updated daily isocenter by adjusting the shape and weight of beam segments in the reference plan. In the ATS workflow, in addition to isocenter shift correction, deformable image registration is also performed to propagate all contours from the reference CT image to the daily MR image. The daily adaptive plan can be optimized by fluence map optimization. Dose calculations are performed on a corresponding synthetic CT that matches the anatomy on the daily MR image.^18^

For each Monaco reference plan, both ATP and ATS plans were generated using diagnostic T2 MRI with a simulated daily positional setup deviation to study the stability of spinal cord dose during the adaptation process. Because these MR images were truncated, all contours including an external body were copied by rigid transformation from the reference CT images. The targets and spinal cord were reviewed after contour propagation from the reference CT to the MRI.

One of our retrospective study on 37 spine SBRT patients who were treated on the TrueBeam Linac showed that the daily setup errors (mean ± SD) were 2.9 ± 3.6 mm (0.8 ± 0.8°), 3.9 ± 3.6 mm (0.7 ± 0.6°) and 3.0 ± 3.0 mm (0.8 ± 0.7°), respectively in the lateral, longitudinal, and vertical directions. The corresponding maximum values were 12.7 mm (3.0°), 11.0 mm (2.9°), and 12.7 (2.6°) mm. Therefore, we chose 10‐mm lateral shift and 2° pitch rotation as a test case of daily setup deviation and applied to all MRI images in the current study. The adaptive plans were compared to the Monaco reference plans after normalizing to the same GTV coverage (V100%) as in the Monaco reference plans. GTVmin and spinal cord D_0.01cc_ values of the adaptive plans were compared by a percent difference with those of the respective Monaco reference plans.

## 3 RESULTS

### 3.1 Patient populations

Ten patients treated between October 2019 and January 2022 were enrolled in the clinical protocol approved by the institutional review board at our institution. Information about these 10 patients, including radiation treatment history, is shown in Table 1. The median GTV was 11.03 cm^3^ (range 2.99–111.50 cm^3^), and the median CTV was 45.23 cm^3^ (range 13.26–229.90 cm^3^).

**TABLE 1:** Patient and treatment information used in the current study

Patient	Previous RT (dose/fractions)	Target	GTV, cm^3^	GTV, Gy	CTV, cm^3^	CTV Rx, Gy	Spinal cord Rx, D_0.01cc_	Clinical SBRT beams
1	T8 (18 Gy/1)	T6‐T7	6.56	27	39.63	21	10	4 VMAT
2	Left chest wall (25 Gy/5)	T10	21.21	27	50.83	24	10	9 IMRT
3	T11 (24 Gy/1)	T9‐T10	8.54	27	71.61	24	10	4 VMAT
4	Right rib (30 Gy/5)	T7‐T9	111.51	27	229.90	24	10	9 IMRT
5	T8 (18 Gy/1)	T9	4.18	27	20.66	21	10	4 VMAT
6	T2‐T4 (24 Gy/1)	T5	13.52	27	29.84	24	10	9 IMRT
7	Mediastinum (52.5 Gy/15)	T7	6.79	27	20.91	24	10	4 VMAT
8	LLL (50 Gy/4)	T8‐T9	67.12	27	180.62	24	14	4 VMAT
9	LLL (60 Gy/30)	T11‐12	33.56	27	117.75	24	14	9 IMRT
10	RUL (60 Gy/30)	T5	2.99	27	13.26	24	14	4 VMAT

Abbreviations: CTV, clinical target volume; D_0.01cc_, maximum dose; GTV, gross tumor volume; IMRT, intensity‐modulated radiotherapy; LLL, left lower lung; RT, radiotherapy; RUL, right upper lung; Rx, prescription; SBRT, stereotactic body radiotherapy; VMAT, volumetric‐modulated arc therapy.

### 3.2 Dosimetric comparison

#### 3.2.1 Target coverage

**FIGURE 1:**
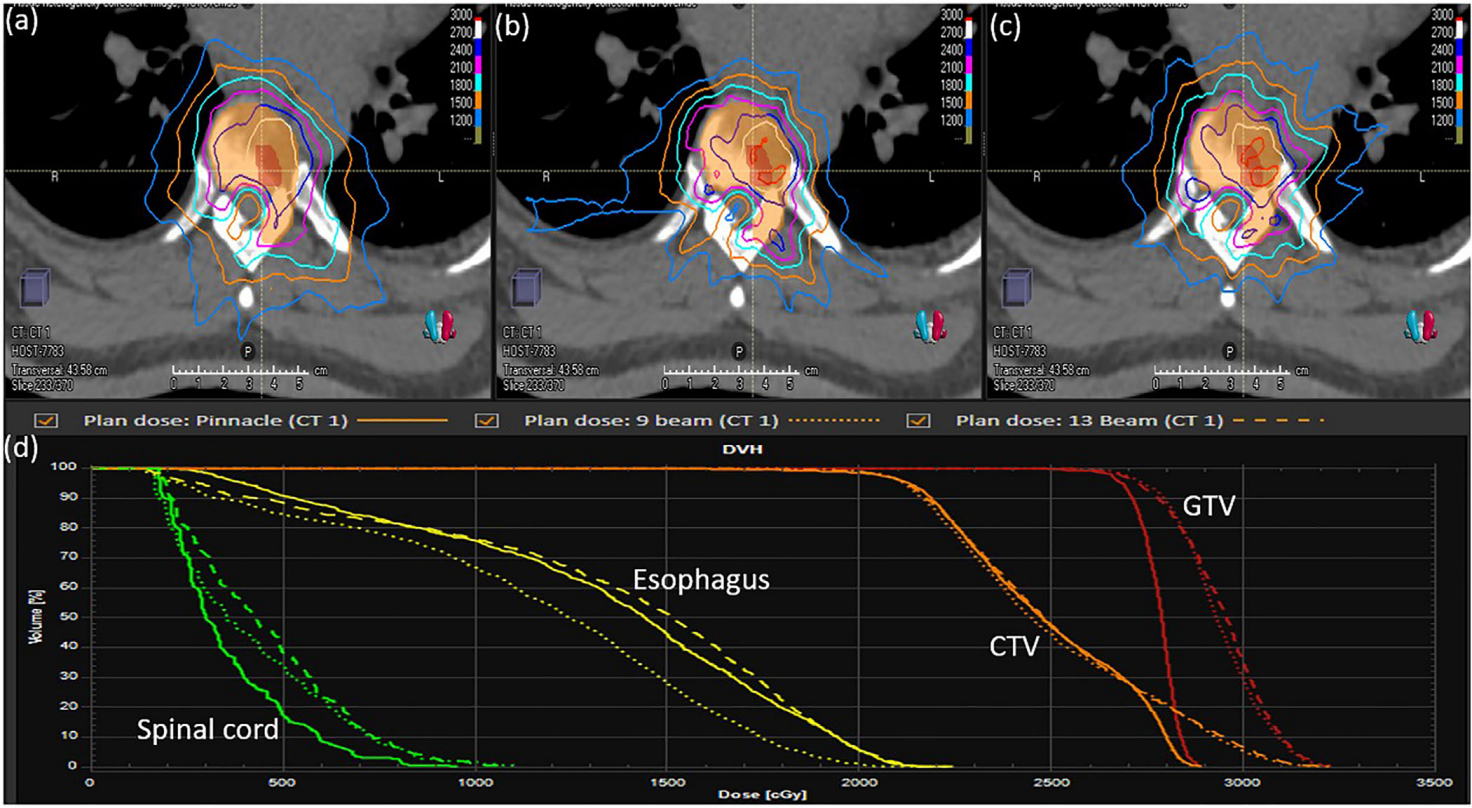
Transverse planar dose distributions and dose‐volume histograms. Planar dose distributions are shown for the Pinnacle plan (a), Monaco 9‐beam plan (b), and Monaco 13‐beam plan (c), along with a comparison of the dose‐volume histograms of the three plans (d). CTV, clinical target volume; GTV, gross tumor volume

Figure 1 shows the representative dose distribution and dose‐volume histogram of the Pinnacle plan, Monaco 9‐beam plan, and Monaco 13‐beam plan. The three plans looked similar (Figure 1a–c), but target doses were much hotter in both Monaco plans, as shown in a dose‐volume histogram comparison (Figure 1d). Table 2 shows a dosimetric comparison of the GTVs and CTVs of the Pinnacle plan, Monaco 9‐beam plan, and Monaco 13‐beam plan, all normalized to have the same GTV coverage (V100%) as in the Pinnacle plan. There were no statistically significant differences in GTV homogeneity index, CTV mean, or gradient index; however, the GTV maximum dose, GTVmin, and GTV mean dose of the Monaco 9‐beam and 13‐beam plans were significantly higher than those in the Pinnacle plan (*p* < 0.05). The GTV PCI and CTV PCI in the Monaco plans were similar to those in the Pinnacle plan. Both the GTV PCI (*p* = 0.018) and CTV PCI (*p* = 0.036) values of the Monaco 13‐beam plans were significantly higher than those observed with the Monaco 9‐beam plans.

**TABLE 2:** Target plan quality index for the three plans (*p*‐values are compared with the Pinnacle plan as a reference)

Plan	GTVmin	GTVmax	GTVmean	GTV HI	CTVmean	GI	GTV PCI	CTV PCI
Pinnacle	15.0 ± 6.7	30.6 ± 1.1	28.1 ± 0.6	1.37 ± 0.28	26.3 ± 0.7	4.7 ± 1.2	0.47 ± 0.13	0.57 ± 0.10
Monaco 9‐beam (*p*‐value)	17.5 ± 6.7 (0.019)	32.1 ± 1.3 (0.012)	29.0 ± 0.9 (0.008)	1.34 ± 0.19 (0.563)	26.8 ± 1.1 (0.174)	4.6 ± 0.8 (0.772)	0.41 ± 0.13 (0.234)	0.57 ± 0.09 (0.965)
Monaco 13‐beam (*p*‐value)	18.0 ± 6.1 (0.003)	31.8 ± 1.1 (0.016)	28.8 ± 0.7 (0.007)	1.33 ± 0.19 (0.424)	26.7 ± 1.0 (0.114)	4.8 ± 0.6 (0.679)	0.45 ± 0.12 (0.721)	0.61 ± 0.08 (0.058)

Abbreviations: CTV, clinical target volume; GI, gradient index; GTV, gross tumor volume; HI, homogeneity index; PCI, Paddick conformity index.

#### 3.2.2 Organ‐at‐risk dose comparisons

**FIGURE 2:**
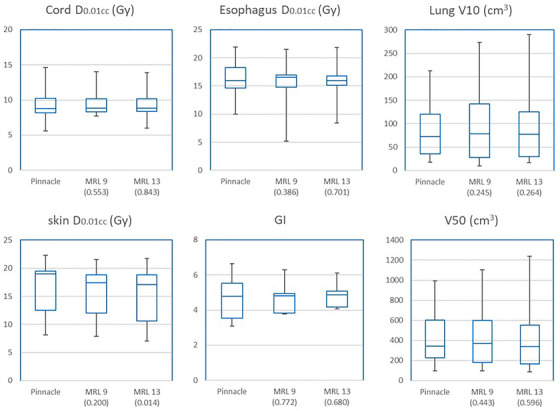
Box graphs showing organ‐at‐risk dose, gradient index, and V50 in Monaco 9‐beam and 13‐beam plans compared with Pinnacle plans. The whiskers show the minimum and maximum values, and the three horizontal lines indicate the twenty‐fifth, fiftieth, and seventy‐fifth percentiles. *p*‐Values are shown in the parentheses. D_0.01cc_, maximum dose; GI, gradient index; MRL, magnetic resonance–guided linear accelerator

Figure 2 shows a dosimetric comparison of organs at risk. No statistically significant differences among the three plans were seen in terms of spinal cord D_0.01cc_, esophagus D_0.01cc_, or lung V10. The skin D_0.01cc_ of the Monaco 13‐beam plan was the lowest among the three plans and was significantly lower than that of the Pinnacle plan (*p* = 0.014). Gradient index and V50 did not statistically differ among the three plans.

### 3.3 Adaptive plan evaluation

**FIGURE 3:**
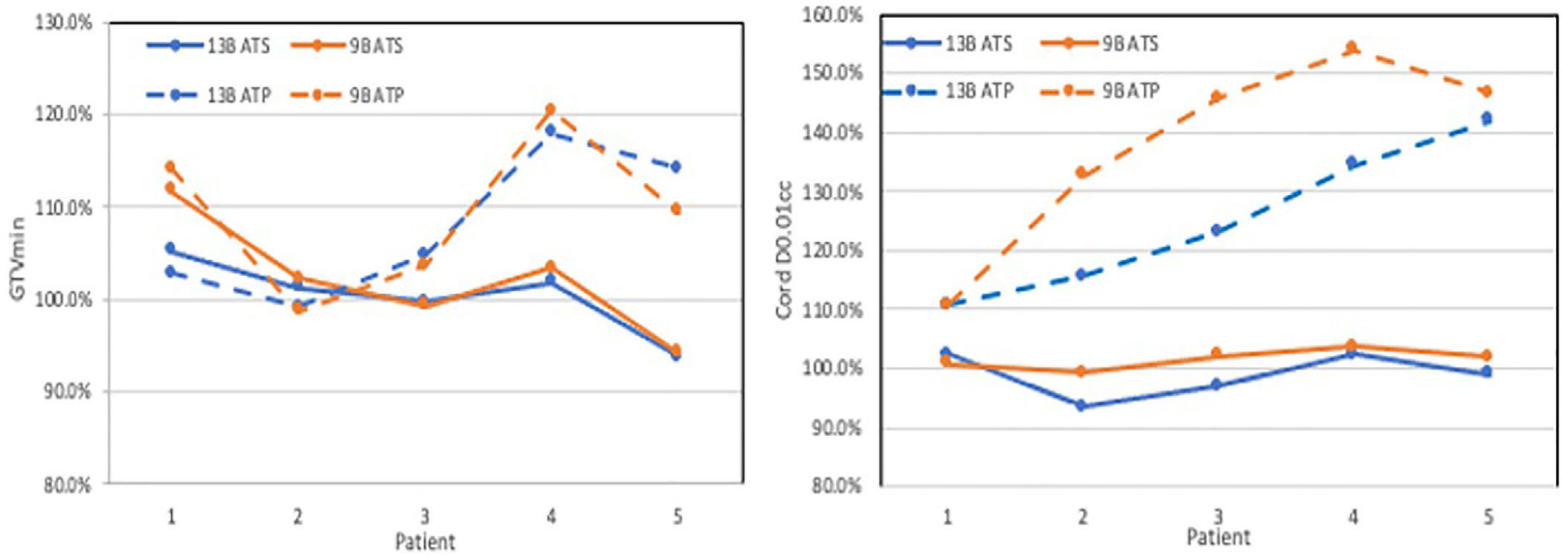
Differences in gross tumor volume (GTV) minimum dose (Left) and spinal cord maximum dose (right) in 13‐beam (13B) and 9‐beam (9B) adaptive plans compared with the respective reference plans for five patients. The patient number 1–5 is not corresponded to Table 1. ATP, adapt‐to‐position workflow (dashed lines); ATS, adapt‐to‐shape workflow (solid lines)

Five of the 10 patients who had MRI images with slice thickness ⩽2 mm were selected for plan adaptation. Figure 3 shows the GTVmin and Cord D_0.01cc_ of the adaptive plans. In the ATP workflow, GTVmin was distributed between 98.7% and 120.4% of the reference plans; however, the spinal cord D_0.01cc_ increased by more than 10% (up to 54%) in all ATP plans, and this trend was worse with the 9‐beam Monaco plans. Three of the five 13‐beam plans and all of the five 9‐beam plans became clinically unacceptable by using the ATP workflow. In the ATS workflow, GTVmin were increased except one patient (−6.0%), and the dose increase of the spinal cord D_0.01cc_ was less than a 3.0% from the Monaco reference plan, and all 9‐ and 13‐beam adaptive plans were clinically acceptable.

## 4 DISCUSSION

The purpose of the current study was to investigate the feasibility of thoracic spine SBRT on the Elekta Unity MRL in patients who have received prior radiotherapy near the spine. Our results show that treatment plans generated on the Monaco TPS can improve the GTVmin with similar target coverage and spinal cord D_0.01cc_ compared with Pinnacle plans and can maintain the consistent plan quality by using the ATS plan adaptation workflow.

The GTVmin values of the Monaco 9‐beam and 13‐beam plans were significantly higher than those of the Pinnacle plans, even with similar doses to the spinal cord. As Lee et al. explained, whereas Pinnacle uses weight‐driven optimization such as Dmax, which penalizes individual hot voxels, Monaco uses constrained optimization that allows a small but controlled number of hot voxels, which often results in improvement of target coverage.^13^ This GTV dose escalation with better visualization of the spinal cord by onboard MRI would fill a need for improved tumor control while safely sparing the spinal cord in cases where the tumor is adjacent to the spinal cord.

We observed the lowest skin D_0.01cc_ in the Monaco 13‐beam plans, and this was different from other authors’ findings.^9^, ^14^, ^15^ It is possible that the multiple encircling beam directions in IMRT plans largely neutralized dose perturbation by the magnetic field.^14^

Although the lung dose (V10) was slightly high with greater variation in Monaco plans, it was still far below the dose constraint, and this is consistent with other studies^9^, ^15^ reporting that the ERE did not induce significant dose effects around air cavities in the patient's body. For two of the patients (patients 4 and 8), the CTV volumes were larger (229.9 cm^3^ and 180.6 cm^3^, respectively), and their lateral extension brought more lung volume close to the target. These patients showed much higher lung V10 in Monaco plans comparing to the Pinnacle plans and thus contributed to the variation of the lung doses.

Although Ding et al.^14^, van de Schoot et al^17^, and Da Silva Mendes et al.^16^ reported an increased low‐dose volume in normal tissue, in our study, low‐dose volumes (V50) were similar (statistically insignificant) among the three plans, even though V50 in Monaco plans shows a greater variation than in Pinnacle plans.

TPS comparison can be challenging owing to several factors. First, different TPSs optimize and convert plans differently. Monte Carlo‐based calculations are expected to obtain higher D_0.01cc_ (or Dmax) when compared with convolution‐based calculations.^13^ Second, the reported differences between Pinnacle plans and Monaco plans in our study are partially due to mechanical differences in treatment machine geometries (larger MLC leaf width and longer SAD) and the presence of the magnetic field in MRL.^2^, ^16^

The MUs of the 13 beam plans and 9 beam plans were 50% and 28% higher than the Pinnacle plans, respectively, and this is consistent with other authors reporting that MRL plans could require >50% more MUs than clinical VMAT plans.^17^, ^19^ However, our mix of 4 IMRT and 6 VMAT Pinnacle plans did not allow full comparison of MUs or delivery time. In a few Monaco plans, achieving target coverage while maintaining the spinal cord dose constraint with D_0.01cc_ ≤120% led to a substantial planning challenge when the GTV wrapped around the spinal cord ≥180° in proximity (≤3 mm from the cord). Monaco planning might require proficient planning skill of planners to overcome this challenge.

## 5 CONCLUSIONS

MRL plans for thoracic spine SBRT in patients who received prior radiation showed safe and favorable tumor dose escalation with spinal cord dose preservation and consistent plan adaptation using the ATS workflow. Careful plan review of hot spots and lung dose would be necessary for safe MRL‐based treatment.

## CONFLICT OF INTEREST

The authors declare that there is no conflict of interest that could be perceived as prejudicing the impartiality of the research reported.

## AUTHOR CONTRIBUTIONS

*Plan generation, data analysis, and writing the manuscript*: Eun Young Han. *Plan review and manuscript review*: Debra N. Yeboa. *Data analysis and manuscript review*: Tina M. Briere. *Data analysis and manuscript review*: Jinzhong Yang. *Plan generation, data analysis, and review the manuscript*: He Wang.
